# Australian Pharmacists’ Perceptions and Practices in Travel Health

**DOI:** 10.3390/pharmacy6030090

**Published:** 2018-08-22

**Authors:** Ian M. Heslop, Richard Speare, Michelle Bellingan, Beverley D. Glass

**Affiliations:** 1 Pharmacy, College of Medicine and Dentistry, James Cook University, Townsville 4811, Australia; michelle.bellingan@jcu.edu.au (M.B.); beverley.glass@jcu.edu.au (B.D.G.); 2 Public Health and Tropical Medicine, College of Public Health, Medical and Veterinary Sciences, James Cook University, Townsville 4811, Australia; richard.speare@jcu.edu.au

**Keywords:** pharmacist, travel health, Australia

## Abstract

Worldwide, pharmacists are playing an increasing role in travel health, although legislation and funding can dictate the nature of this role, which varies from country to country. The aim of this study was to explore the current and potential future practices in travel health for pharmacists in Australia, as well as the perceived barriers, including training needs, for the provision of services. A survey was developed and participation was sought from a representative sample of Australian pharmacists, with descriptive statistics calculated to summarise the frequency of responses. A total of 255 participants, predominantly female (69%), below 50 years (75%) and registered less than 30 years completed the survey. Although over two-thirds (68%) provided travel-related advice in their current practice, the frequency of advice provision was low (less than 2 travellers per week) and limited to responding to travellers questions. Although Australian pharmacists are currently unable to administer travel vaccines and prescription only medications without prescription, they still consider travel health to be an appropriate role and that their clients would seek travel health advice from pharmacies if offered. Currently, key roles for Australian pharmacists are advising travellers who do not seek advice from other practitioners, reinforcing the advice of other health practitioners and referring travellers needing vaccinations and antimalarials. In order to expand these services, the barriers of workload, time, staffing and the need for training in travel health need to be addressed. In summary, the travel health services provided by pharmacies in Australia still have a way to go before they match the services offered by pharmacies in some other countries, however Australian pharmacist are keen to further develop their role in this area.

## 1. Introduction

International travel is on the increase, especially to destinations in Asia, Africa and other emerging economies and the associated health risks of these destinations highlights the need for pre-travel health consultations [1,2]. Despite this, many travellers do not obtain pre-travel health advice before travelling overseas and, those who do, mainly seek advice from their general practitioner, a travel health clinic or specialist, or the internet [3,4,5,6,7]. Pharmacies do offer some travel health services, although the type and level of these services may vary, with pharmacy-run travel health services enjoying limited patronage. However, it has been suggested that pharmacies are perhaps an underutilised resource and that their accessibility, convenient location and the trust placed in pharmacists by the public make them an appropriate source of pre-travel health information [8,9,10]. 

A comprehensive assessment, analysis of risk and tailored counselling are all important for the pharmacist to deliver evidenced–based pre-travel health consultations. Current practice for pharmacists in travel health may vary significantly from country to country and can be attributed to legislative differences which may often not allow pharmacists, for example in South Africa, to prescribe or dispense medications without prescriptions as well as administer travel vaccines [11]. However, the scope of practice for South African pharmacists has expanded as a result of the down scheduling of some antimalarial drugs [11]. Similarly, within the UK, changes in the legislation providing pharmacists with a wider scope of practice to supply some prescription only medications combined with a 5% increase in the number of Britons travelling aboard has led to pharmacists having a greater role within a nationally funded travel health service [12]. Some of these current initiatives have been well received in the UK, with patients reporting that a pharmacy-run travel health service both met their needs and provided value for money [13]. 

Bascom et al. however reported that the overall confidence in providing travel health advice in a group of pharmacists surveyed in Alberta, Canada was low, with incomplete knowledge possibly impacting their ability to this provide advice. Although this study was limited by the sample size, it is suggested that this barrier could be addressed by training programs, both at undergraduate level and with continuing professional education [14]. The findings of this study were revisited by Houle, who found that pharmacists were confident in areas most commonly seen in community pharmacy practice, with 67% confident that they had the ability to source the required information. This again highlights the need for inclusion of travel health into university curricula to expand the scope of practice to include these new practice opportunities. 

Houle, in a review on current and future prospects for travel health services, indicated the role that pharmacist have been playing in ensuring the cold chain for vaccinations has placed them in a good position to extend their scope of practice from administering just the influenza vaccine to travel vaccines [2]. This has already been adopted in some Canadian jurisdictions [2]. There are a number of different pharmacist prescribing models across the UK, USA and Canada, with pharmacists in all countries traditionally providing non-prescription drugs for traveller’s diarrhoea, motion sickness and sun and insect bite protection [2,10,12,13,14,15,16,17]. The well-established medication documentation systems of pharmacies could also play a role in assisting patients to maintain documentation on their vaccination history and while travel health consultations focus largely on infectious diseases, the impact of non-infectious causes of morbidity and motility during travel cannot be overemphasised and this again presents pharmacists with an opportunity for which they are already trained [12,13,14,15,16,17].

The aim of this study was thus to examine both the current practices and opportunities for future practice of Australian pharmacists in the provision of travel health services. Their views regarding some of the barriers to implementation of these services and the need for training will also be explored.

## 2. Materials and Methods 

### 2.1. Study Design and Participants

This study involved a cross-sectional survey of Australian pharmacists. The questionnaire was formatted into an electronic e-survey using SurveyMonkey^®^. Invitations to participate and hyperlinks to the questionnaire and participant information leaflet, were then e-mailed to all members of the Pharmaceutical Society of Australia in a weekly newsletter. In addition, the self-completion questionnaire was also formatted into a postal survey using Microsoft Word^®^ and posted to a representative, stratified sample of 600 Australian community pharmacies. This sample was drawn from the estimated 7600 pharmacy businesses listed in the then current Yellow Pages^®^ Business Directory for Australia using a systematic random sampling technique, ensuring a representative sample. The e-survey was open for a 6-week period from late March 2009 and the postal survey was open for a 6-week period from early May 2009. 

### 2.2. Questionnaire Design and Testing

A self-completion questionnaire, consisting of a combination of 44 multiple choice questions (MCQs), multiple answer questions (MAQs), open answer and rating scale questions (using 5 point Likert scales) was designed to meet the objectives of the study. Questions were divided into 3 main sections; Demographics, Current travel health services and Perceptions of current and future travel services. To ensure the validity and reliability and to reduce bias and to allow comparison with other studies, some of the questions used in the self-completion questionnaire were based on similar questions used in other surveys [18,19]. In addition, before the questionnaire was distributed, it was pre-tested by a group of 5 pharmacists for understanding, readability and to ensure a timely completion. Only minor grammatical changes were then made prior to distribution. 

### 2.3. Data Analysis

The responses to the e-survey and postal surveys were entered into Microsoft Excel^®^ spreadsheets and the IBM^®^ SPSS Statistics Package^®^ (Version 22) was used for statistical analyses. 

### 2.4. Ethical Considerations

Ethical approval for the study was granted by the James Cook University Human Research and Ethics Committee (Approval No: H3182) and approval to send a postal survey to the community pharmacies was obtained from the Survey Approval Program of the Pharmacy Guild of Australia (Approval No: 755). 

## 3. Results

### 3.1. Respondent Characteristics

A total of 255 participants completed the survey. Participants were predominantly female (69%, 176/255), below the age of 50 years (74.5%, 190/255) and registered less than 30 years as a pharmacist (80.4%, 205/255). Most resided in metropolitan areas or capital cities (77.3%, 197/255) and were working in full-time positions (69.4%, 177/255), predominantly in community pharmacy (78.4%, 121/255). The majority of respondents had standard entry level pharmacy qualifications (82.7%, 211/255) and some had additional postgraduate qualifications including 9.8% (25/225) with postgraduate certificates, 6.7% (17/255) with a Master’s degree and 0.8% (2/255) with doctorates. All Australian States and Territories were represented in the sample. 

### 3.2. Current Practices

Over two-thirds of respondents (68.2%, 174/255) provided travel-related advice or services. However, their travel health workload was generally low, with the majority advising less than two travellers per week (69%, 120/174) and/or spending less than one hour per week on the provision of these services (83.9%, 146/174). The respondents reported that they commonly advise Australian travellers aged either below the age of 30 (56.9%, 99/174) or above the age of 50 (47.7%, 83/174), travelling for leisure (98.9%, 172/174), business (51.2%, 89/174) or were visiting friends and relatives (51.7%, 90/174) and to destinations in mainly in Southeast Asia (92%, 160/174), Western Europe (54%, 94/174) or Oceania (28.2%, 49/174) regions. 

When questioned about the type and level of travel health service offered, over a third of respondents (34.5%, 60/174) reported that they only responded to travellers’ questions and did not perform formal pre-travel health risk assessments, although 64.5% (112/174) of respondents reported that they did ask the traveller questions about their itinerary and medical history. Only 2 respondents (1.1%) completed full, formal pre-travel health risk assessments for their clients. In addition, respondents were asked to rate how often they counselled travellers on a range of 26 recommended travel health topics using a 5-point Likert scale. The mean ratings were calculated and are presented in Table 1. The majority of respondents (59.8%, 104/174) reported that they counselled travellers using a combination of written and verbal information and a similar number reported that they used generic drug information resources such as the Australian Medicines Handbook and the Australian Immunisation Handbook to respond to travellers’ questions, whereas few reported that they used more travel-specific websites such as MASTA (34.5%, 60/174) and Travax (19%, 33/174).

### 3.3. Future Practices

**Figure 1:**
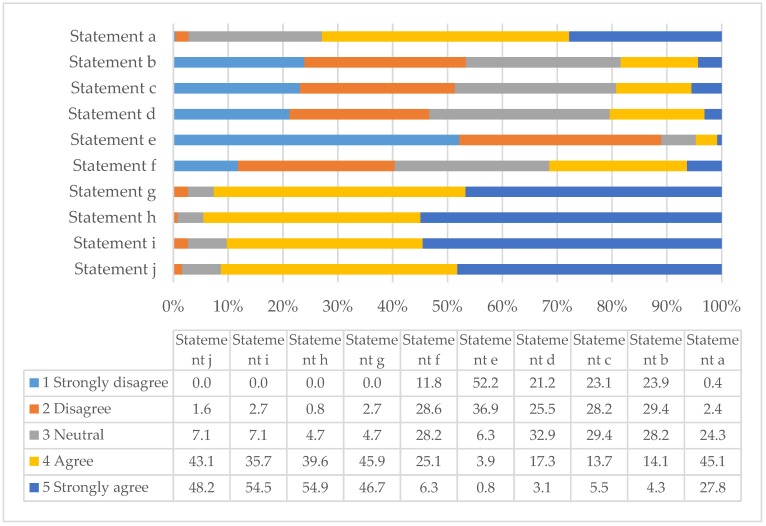
Percentage ratings of how frequently pharmacists agreed or disagreed to statements relating to the current or future roles of pharmacists in the area of travel health (n = 255).

By rating their level of agreement or disagreement to standard statements with 5-point Likert scales, respondents gave their views regarding the current and future roles of Australian pharmacists in the area of travel health, potential barriers to service development and the training needs of pharmacists. Table 2 gives the respondents’ average rating to each statement examining their views of travel health as an appropriate current and potential future role for Australian pharmacists in travel health. Table 2 also acts as the key for Figure 1, which summarises the percentage of the respondents who chose a particular rating for each statement. 

Nearly 90% of respondents disagreed/strongly disagreed with statement *e* (average rating 2.0) thereby demonstrating that they consider travel health to be an appropriate role for pharmacists. In addition, because 72.9% of respondents agreed/strongly agreed with statement *a* (average rating 4.0), they also feel that travellers would support pharmacist-run travel health services. At the time of the survey it was uncommon for Australian pharmacies to offer vaccination services and they were and still are, unable to supply prescription only medications without a prescription from an appropriate prescriber. However, it appears that the respondents do not see this as a major barrier to travel health service development (53.3% and 51.3% of respondents disagreed/strongly disagreed with statements *b* and *c* respectively). In addition, although the safe dispensing and supply of medications is recognised as a core function of pharmacists in the healthcare system, responses suggest that respondents were divided as to whether this should be their only function in the area of travel health (40.4% disagreed/strongly disagreed to statement *f*, whereas, 31.4% agreed/strongly agreed and 28.2% appeared neutral). Responses suggest that they felt more strongly that suitable roles for pharmacists in travel health included giving travel health advice to travellers who would not normally obtain pre-travel advice from their doctor (92.6% agreed/strongly agreed with statement *g*), supplementing or reinforcing the advice given by other practitioners, advising on travel-related health issues that may not have been covered by their doctor (91.3% agreed/strongly agreed with statement *j*) and referring some travellers back to their doctor if they are visiting certain destinations, perhaps for vaccinations and antimalarials or other medications (94.5% agreed/strongly agreed with statement *h*). Finally, they agreed that pharmacists have a role in the supply of traveller’s first aid kits and advising on their contents (90.2% agreed/strongly agreed with statement *i*).

**Figure 2:**
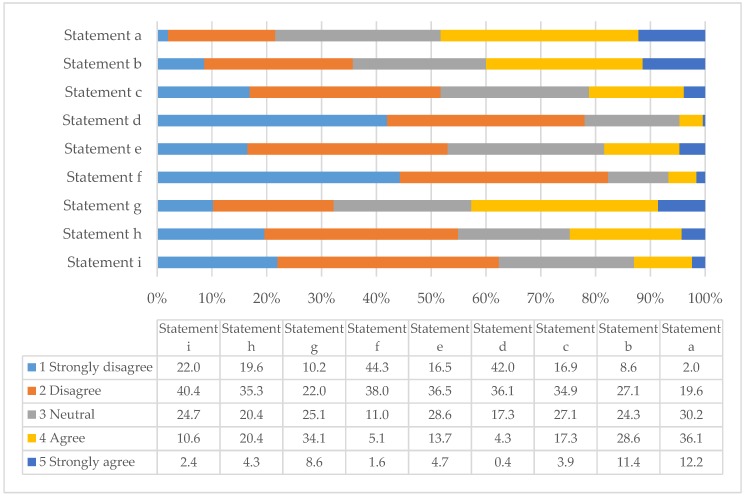
Percentage ratings of how frequently pharmacists agreed or disagreed to statements relating to potential barriers that may limit of slow the development of pharmacists with regard to travel health (n = 255).

Likewise, by rating their level of agreement/disagreement to standard statements using a 5-point Likert scale, respondents also gave their views relating to potential barriers to the development of pharmacist-run travel health services. Table 3 gives the respondents’ average rating to each statement and also acts as the key for Figure 2 which summarises the percentage of the respondents who chose a particular rating for each statement. 

Although respondents were very interested in providing travel health services (82.3% disagreed/strongly disagreed with statement *f*), they did recognise both time and staffing to be potential barriers (48.3% and 40% agreed/strongly agreed to statements *a* and *b* respectively). However, responses were divided because 30.2% of respondents were also neutral to statement a (time) and 35.7% disagreed/strongly disagreed with statement *b* (staffing). One criticism of extended pharmacy services is that they are often undertaken by pharmacy assistants and not qualified pharmacists [20], however the respondents do not appear to view travel health as a potential role for pharmacy assistants (only 21.2% agreed/strongly agreed with statement *c*). Importantly, the respondents felt that travel health services from pharmacies could be profitable and, again, that the inability to supply prescription only medications without prescription would not adversely affect the viability of the service (53% and 54.9% of respondents disagreed/strongly disagreed with statements *e* and *h* respectively). It appears that respondents did not expect antipathy from the medical profession and, that even if this was the case, it would not prevent them from developing travel health services (62.4% disagreed/strongly disagreed with statement *i*).

Finally, although 68.2% (174/255) of the respondents provide some level of travel health service, the vast majority of respondents (96.9%, 247/255) had not received any formal travel health training. However, 42.7% of respondents did recognise that they require further training, if they wish to provide quality travel health services and the majority would wish that training to be accredited either by a pharmacy (41.6%, 106/255) or travel medicine (45.1%, 115/255) professional body. They would also prefer the training to be available either online (40%, 102/255) or using a combined on-line and block mode delivery method (52.5%, 134/255).

## 4. Discussion

At the time of the survey, few Australian pharmacies offered immunisation services and were and are currently, unable to supply antibiotics and antimalarial medications without a prescription. However, a large number of respondents offered some form of travel health service and although most only responded to simple travel health enquires instigated by the traveller, a small number did offer comprehensive pre-travel health risk assessments for their clients. The travel health workload in all pharmacies appeared low. In contrast, although many of the reported international pharmacy-run travel health services appear to offer fully comprehensive services, supported with standard questionnaires and interview schedules to aid the assessment of travellers [13,15,17,21,22,23], their workload is also often low and comparable with that of this study. Kodkani et al. [18] also reported a variation in pharmacy travel health workload in Switzerland, with 8% of respondents giving frequent advice (more than 5 times per week) and 10% giving infrequent advice (less than 6 times per year). However, the majority of respondents (56%) in the Kodkani study only gave travel health advice to 2–3 clients per month. Likewise, Teodosio et al. [19] reported that 87.6% of Portuguese pharmacies in their study only advised up to 3 travellers per month. 

Many of the respondents only discussed a limited range of health topics with their clients. However, the top ten topics that respondents most frequently discussed aligned with the recommended travel health counselling topics suggested by Spira [24]. These are also the topics of most interest to travellers, such as vaccinations and malaria chemoprophylaxis and issues relating to common travel-related conditions, such as Traveller’s diarrhoea. However, the list also includes areas highlighted as key areas for pharmacist input, such as medication management and travel first aid kits. Topics rarely discussed with travellers included more specialised travel health situations, such as diving-related illness and acute mountain sickness. In addition, pharmacists rarely advised travellers about some relatively common travel health situations and issues, such as the risk or prevention of sexually transmitted diseases and accidents and how to obtain medical care overseas. There are many reasons why pharmacists do or do not counsel travellers on some topics with time limitations being a major factor, as is the perception by pharmacists as to their role, which may not extend to travel insurance, the prevention of accidents or diving and mountaineering-related issues. Other studies have examined the advice given to travellers by both doctors and pharmacists and some have found some deficiencies or omissions in the advice given [18,19,25,26]. For example, it was found that although high numbers of Swiss and German general practitioners (GPs) regularly gave travel health advice, many did not provide correct recommendations for vaccinations and malaria chemoprophylaxis for common tropical destinations [26]. Likewise, another study also found that some GPs also gave a limited range of pre-travel health advice and that, for example, over 50% of the GPs surveyed did not give travellers pre-travel advice on the risk and prevention of sexually transmitted diseases [27].

Travel health is a rapidly changing field and travel health providers must keep abreast of these changes, if they are to provide the most accurate and up to date information [28]. Some studies have examined the information resources used by other travel health providers such as GPs [26,28,29]. However, there appears to be little known about the information resources used by Australian pharmacists in the provision of travel health advice. It appears that the respondents tend to use more generic drug information resources and few specialist travel health information resources. However, the publications used are readily accessible, regularly up-dated and are fairly economical in price. Hatz et al. [26] found that Swiss GPs tend to prefer national resources and guides and Leggat and Seelan [28] also found that the Australian Immunisation Handbook was a commonly used resource by Australian GPs. Few of the respondents mentioned that they referred to peer reviewed journals for information, perhaps due to a lack of accessibility. This concurs with findings from Australian GPs by Leggat and Seelan [28]. 

The restrictions placed on Australian pharmacists offering vaccination services and to supply prescription only medications without a prescription could have been perceived as a barrier to travel health service development, however the results of this study show that this does not appear to be the case. In the UK and USA, the ability to offer vaccinations in pharmacies without prescription has been an important enabling factor for the development of pharmacy-run travel health services [9,13,30]. Therefore, as pharmacy immunisation services are now more common, it would be interesting to reinvestigate the views of Australian pharmacists about pharmacy-run travel health immunisation services in more detail. Inadequate staffing levels, time, lack of training and antipathy with other health professions are often listed as potential barriers to the development of extended services by pharmacists [31]. However, in this study antipathy from other health professionals did not appear to be a major concern for respondents and although staffing and time were reported as potential barriers, responses were also divided.

Finally, the fact that many respondents self-recognised the need for further training in order to deliver high quality travel health services is consistent with the findings of other studies [2,20] who also reported that the vast majority of respondents in their study (93.2%) had no training in travel medicine, however they did note that 77.9% did attempt to stay informed or be updated. Currently, there are no pharmacist-specific, accredited travel health training programs available for pharmacists in Australia. Therefore, this is an ideal opportunity for an Australian pharmacy professional body to work collaboratively with a peak travel health body, such as the International Society of Travel Medicine, in order develop a pharmacist-specific training program for Australian pharmacists to further progress the role of Australian pharmacists in this specialty area.

## 5. Conclusions

Findings from this study confirm that travel health is an appropriate role for Australian pharmacists and that their clients would seek travel health advice from pharmacies if offered. Therefore, there is consensus that expanding current practices from simple reactive services responding to travel-related enquiries to comprehensive pre-travel health risk assessments is an opportunity for future practice. Overcoming barriers of workload, time and the need for training will bring the Australian pharmacists into line with international practice and provide better outcomes for Australians travelling overseas.

## Figures and Tables

**Table 1:** Average ratings for how frequently respondents advise travellers about common travel-related health topics (In order. Top 10 topics shaded) (n = 174).

Counselling Topic	Average Rating (Scale 1–5)
Treatment of diarrhoeal diseases	4.2
Prevention of mosquito and other insect bites	4.2
Safe food and water consumption	4.0
The need for antimalarial chemoprophylaxis	3.9
Travelling with medications for chronic conditions	3.9
Vaccinations needed for the traveller’s destination	3.8
Risk and prevention of deep vein thrombosis	3.3
Dealing with pre-existing conditions (e.g., diabetes) whilst travelling	3.3
The recommended contents of a first aid kit	3.3
Travelling with a medical or first aid kit	3.2
Tropical diseases at their destination	2.9
Methods of water purification	2.9
The need for early diagnosis and treatment of malaria	2.8
Health issues of travelling with children	2.8
Altering dosages of medications when travelling through multiple time zones	2.7
Prevention and treatment of jet leg	2.6
Current disease outbreaks at their destination	2.5
Need for travel medical insurance	2.4
Health issues of travelling whilst pregnant	2.2
Risk and prevention of accidents whilst overseas	2.0
Risk and prevention of sexually transmitted diseases	2.0
How to obtain medical care whilst overseas	2.0
Prevention and treatment of acute mountain sickness	2.0
Safe alcohol and drug consumption whilst overseas	1.9
Issues regarding personal safety and crime prevention	1.9
Prevention and treatment of diving-related illnesses	1.7

Scale used: 1-Never advise, 2-Rarely advise, 3-Occasionally advise, 4-Frequently advise, 5-Always advise.

**Table 2:** Average ratings for how frequently pharmacists agreed or disagreed to statements relating to the current or future roles of pharmacists in the area of travel health (n = 255).

Statement	Average Rating (Scale 1–5)
a. Travelers want pharmacists to offer travel health services	4.0
b. Pharmacists cannot offer adequate travel health services as they cannot administer vaccines	2.0
c. Pharmacists cannot offer adequate travel health services as they cannot supply S4 medications without prescription	3.0
d. Offering travel health services would cause antipathy with the medical profession	3.0
e. Travel health is not an appropriate role for pharmacists	2.0
f. The most appropriate role for pharmacists in travel health is to check the appropriateness of medications prescribed for the traveller	2.9
g. The pharmacist has a role advising travellers who would not normally visit a doctor before travelling on travel-related health issues	4.0
h. The pharmacist has a role advising travellers whether to seek medical advice before visiting certain destinations	4.0
i. The pharmacist can adequately advise the traveller on items to place in a first aid kit when travelling to remote destinations	4.4

Scale used: 1-strongly disagree, 2-disagree, 3-neutral (neither agree or disagree), 4-agree, 5-strongly agree.

**Table 3:** Average ratings for how frequently pharmacists agreed or disagreed to statements relating to potential barriers that may limit or slow the development of pharmacists’ roles with regard to travel health (n = 255).

Statement	Average Rating (Scale 1–5)
a. The average community pharmacist would not have enough time to provide quality travel health services	3.4
b. My pharmacy has inadequate staffing levels to provide quality travel health services	3.0
c. Pharmacy assistants could advise travellers on travel-related health issues	3.0
d. Travelers do not want pharmacies to offer travel health services	2.0
e. Travel health services would not be profitable for pharmacies	3.0
f. I am not interested in providing travel health services	1.8
g. Pharmacists are inadequately trained to provide travel health services	3.0
h. The inability to supply S4 medications without prescription would make travel health services unviable from pharmacies	3.0
i. Perceived antipathy from other health professionals would stop me developing travel health services	2.3

Scale used: 1-strongly disagree, 2-disagree, 3-neutral (neither agree or disagree), 4-agree, 5-strongly agree.
